# Evaluating noninvasive methods for estimating cestode prevalence in a wild carnivore population

**DOI:** 10.1371/journal.pone.0277420

**Published:** 2022-11-15

**Authors:** Ellen E. Brandell, Madeline K. Jackson, Paul C. Cross, Antoinette J. Piaggio, Daniel R. Taylor, Douglas W. Smith, Belgees Boufana, Daniel R. Stahler, Peter J. Hudson

**Affiliations:** 1 Center for Infectious Disease Dynamics, Department of Biology, Huck Institutes of Life Sciences, Pennsylvania State University, University Park, PA, United States of America; 2 Yellowstone Center for Resources, Yellowstone National Park, WY, United States of America; 3 U.S. Geological Survey, Northern Rocky Mountain Science Center, Bozeman, MT, United States of America; 4 National Wildlife Research Center, U.S. Department of Agriculture, Animal and Plant Health Inspection Service, Wildlife Services, Fort Collins, CO, United States of America; 5 National Wildlife Management Centre, National Reference Laboratory for Parasites (*Trichinella* and *Echinococcus*), Animal and Plant Health Agency, York, United Kingdom; Beni Suef University Faculty of Veterinary Medicine, EGYPT

## Abstract

Helminth infections are cryptic and can be difficult to study in wildlife species. Helminth research in wildlife hosts has historically required invasive animal handling and necropsy, while results from noninvasive parasite research, like scat analysis, may not be possible at the helminth species or individual host levels. To increase the utility of noninvasive sampling, individual hosts can be identified by applying molecular methods. This allows for longitudinal sampling of known hosts and can be paired with individual-level covariates. Here we evaluate a combination of methods and existing long-term monitoring data to identify patterns of cestode infections in gray wolves in Yellowstone National Park. Our goals were: (1) Identify the species and apparent prevalence of cestodes infecting Yellowstone wolves; (2) Assess the relationships between wolf biological and social characteristics and cestode infections; (3) Examine how wolf samples were affected by environmental conditions with respect to the success of individual genotyping. We collected over 200 wolf scats from 2018–2020 and conducted laboratory analyses including individual wolf genotyping, sex identification, cestode identification, and fecal glucocorticoid measurements. Wolf genotyping success rate was 45%, which was higher in the winter but decreased with higher precipitation and as more time elapsed between scat deposit and collection. One cestode species was detected in 28% of all fecal samples, and 38% of known individuals. The most common infection was *Echinococcus granulosus sensu lato* (primarily *E*. *canadensis*). Adult wolves had 4x greater odds of having a cestode infection than pups, as well as wolves sampled in the winter. Our methods provide an alternative approach to estimate cestode prevalence and to linking parasites to known individuals in a wild host system, but may be most useful when employed in existing study systems and when field collections are designed to minimize the time between fecal deposition and collection.

## Introduction

Helminth infections (i.e., cestodes, nematodes, trematodes) are ubiquitous in free-living wildlife populations and can affect host behavior, survival, reproduction, and population dynamics [1–4]. For instance, nematode infections in hosts such as spotted hyenas (*Crocuta crocuta*) and South American fur seals (*Arctocephalus australis*) led to increased juvenile mortality [5, 6]. High helminth intensities can also make prey more susceptible to predators [7, 8] and can interact with host stressors like co-infections and thermoregulation to cause morbidity by altering host energy [2, 9, 10]. However, the distribution and consequences of intestinal parasite infections remains unknown for some host species (e.g., [11]) and, especially, carnivores (but see [12]).

Collecting samples and studying intestinal parasite infections in carnivore hosts can be challenging. Samples are often obtained by trapping or hunting hosts and performing necropsies (e.g., [13, 14]), which involves logistical and ethical considerations. These studies provide information about parasite presence and distribution, but do not provide longitudinal infection data within a host. Noninvasive techniques where host scat is collected and analyzed for parasite eggs, oocysts, or larvae has been used to identify helminths in numerous wild carnivore species, including in protected areas where invasive and lethal practices may be highly regulated or prohibited [15]. These samples can also be used to obtain DNA of the host for species, individual, and sex identification, and to evaluate relationships among hosts [16–18].

Here we used a combination of methods to analyze the cestode communities infecting gray wolves (*Canis lupus*) in Yellowstone National Park, USA. We aimed to build on previous and current methods to leverage information from scat samples in an intensively monitored wolf population. A few studies have identified helminth infections in wolves across their range using noninvasive methods [19, 20], yet samples are often analyzed at the group-level [15, 21], or at the individual-level but lack individual-level covariates [22]; therefore, inference about infections in individual wolves is limited. Additionally, molecular methods are required for identifying parasite taxa with similar egg morphology, such as taeniid cestodes [23, 24]. For these taxa, parasite species that are morphologically similar may have very different consequences for hosts–e.g., the eggs of *Echinococcus multilocularis* and *Taenia pisiformi*s are morphologically identical, yet *E*. *multilocularis* is known to be zoonotic while *T*. *pisiformi*s is not–thus distinguishing among species can be essential. Using noninvasively collected DNA is a common tool in wolf management and thus provides an easily paired method for species confirmation and individual relatedness along with noninvasive parasitology (e.g., [17, 25–27]).

We used the Yellowstone wolf population to evaluate the performance of a combination of noninvasive parasitology and host genotyping (invasive and noninvasive) at the individual wolf level, which we paired with intensive observational data, to analyze associations between wolf social and biological characteristics and helminth infections. Specifically, we focused on the cestode communities of wolves because cestodes (*Taeniidae*) are the most common helminth taxa infecting Yellowstone wolves [15], and *Echinococcus granulosus* has previously been detected [28]. Cestodes are common intestinal, parasitic tapeworms–adults usually occupy vertebrate digestive tracts, and larvae occupy bodies of vertebrates or invertebrates [29]. Wolves, a definitive host, play a crucial role in the maintenance and transmission of *Taeniidae* [22, 30, 31], although prevalence in wolves is conditional on their major prey species (i.e., intermediate hosts) [32]. Importantly, certain cestode species, such as *E*. *granulosus* and *E*. *multilocularis*, are zoonotic [30, 31] and infect and cause disease in livestock [22, 33, 34]. For these reasons, monitoring and preventive medicine development occurs globally [35, 36]).

Helminth infections are heterogeneously aggregated within host populations due to variation in exposure and host susceptibility, which can vary with host behavior [37], physiology (e.g., sex and stress [38, 39]), social rank [39, 40], and diet [41, 42]. Cestode infections vary seasonally corresponding with cyst development and population cycles in intermediate hosts [43]. In addition to infection heterogeneities due to host characteristics, scat samples vary in quality due to environmental degradation that occurs upon deposition. Quality and quantity of DNA from fecal samples is critical to successful host genotyping, and understanding the environmental effects on sample quality is important when designing or evaluating noninvasive DNA studies (e.g., [17, 44, 45]).

**Fig 1:**
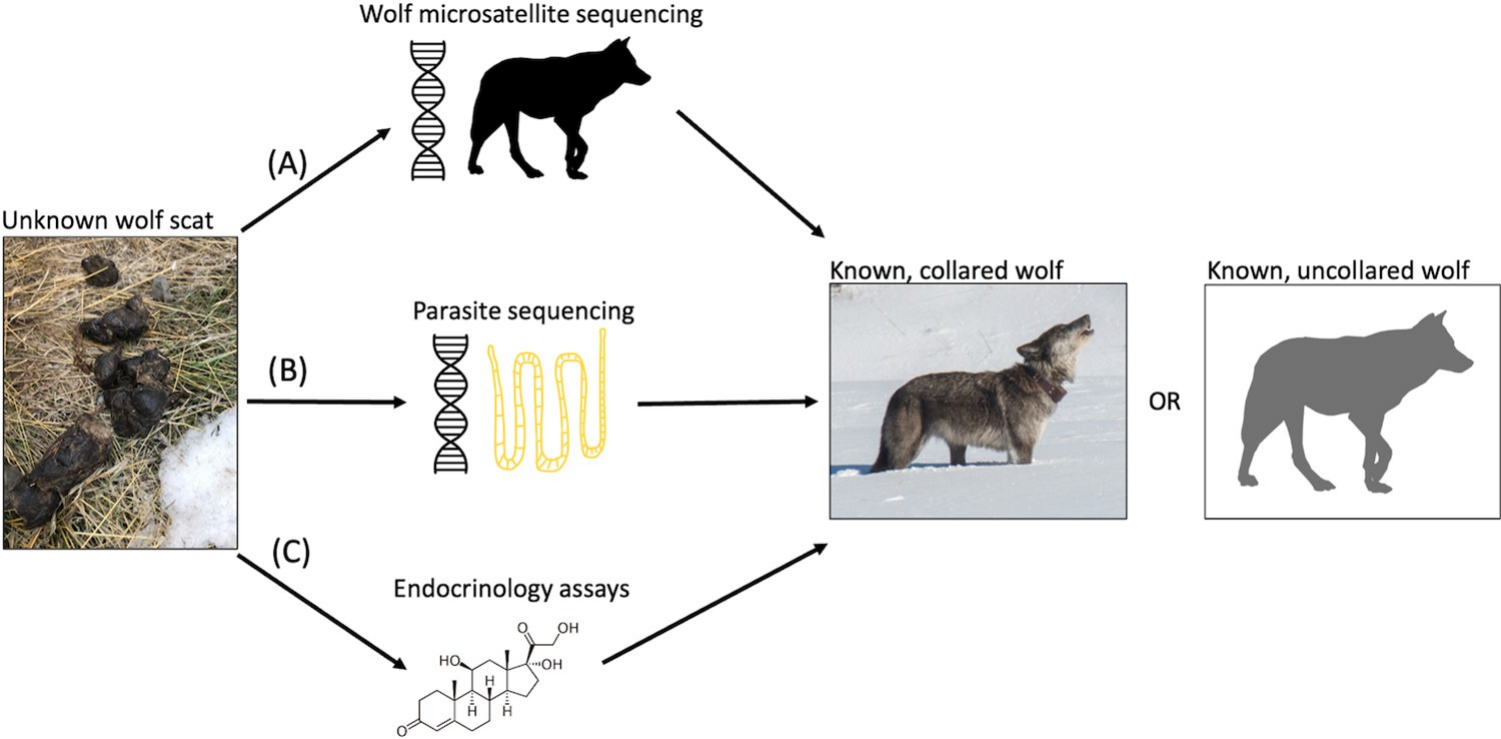
Flow diagram of our methods for analyzing wolf scats. Scats were collected in the field (left), and then processed in three ways: (A) wolf DNA was extracted, genotyped based on microsatellites, and sex was identified; (B) cestode DNA was extracted and genotyped to species; and (C) metabolites of fecal glucocorticoids (molecule shown) were quantified, resulting in a measure of stress. Scat analyses were then matched (right) to known collared wolves, who were monitored throughout their lives, or uncollared wolves. Photo credit: Ellen E. Brandell (wolf scat, left), Jort Vanderveen (collared wolf, right).

Our goals were three-fold: (1) Identify the species and apparent prevalence of cestodes infecting Yellowstone wolves; (2) Assess the relationship between wolf biological and social characteristics and cestode infections; (3) Examine how wolf samples were affected by environmental conditions with respect to the success of individual genotyping. We achieved these goals using three years of wolf scat collection (2018–2020), a combination of field and laboratory methods (Fig 1), and statistical analyses. Finally, we evaluate our approach for future work and its applications in other systems.

## Materials and methods

### Study area and sample collection

We focused on the northern portion of Yellowstone National Park (Montana and Wyoming), where wolves were monitored year-round and wolf density was high (mean 58 wolves/1000-km^2^). Northern Yellowstone National Park is mountainous (ranging approximately 1500–2200 m) and is characterized primarily by steppe, grassland, and coniferous forests, with relatively few wetland areas. Wolves are highly social species that reside in packs. Approximately 30% of the northern Yellowstone wolf population is equipped with GPS radio collars at any given time, and wolves and packs are monitored for survival, reproduction, predation, and behavioral purposes. Common prey species, and therefore potential intermediate hosts [29, 46, 47], in northern Yellowstone National Park included elk (*Cervus canadensis*), deer (*Odocoileus virginianus/hemionus*), and small mammals (e.g., *Spermophilus armatus*, *Lepus americanus*).

Scat samples were collected from 2018 through 2020 during four primary periods: early winter (30 days: November 15-December 15), late winter (30 days: March 1-March 30), summer (~75 days: May 15-July 31), and following denning (August-October). Scats were collected from four packs: Junction Butte, 8 Mile, Wapiti Lake, and Crevice Lake (including 1005F Group and Carnelian Creek packs). We used GPS data from wolves annually live captured and sampled (including blood serum) following protocols approved by NPS Institutional Animal Care and Use Committee (IACUC IMR_YELL_Smith_wolves_2012). Each captured wolf is identified with a unique wolf ID. We prioritized areas where we would most likely encounter wolf scat: prey carcass sites, GPS clusters, and vacated dens. GPS clusters are spatio-temporal GPS points where wolves (typically most or all of a pack) were located for an extended period of time [48]. With this design, scats were collected, on average, 17 days following deposit (range 1–75 days). Dens were identified by GPS points and visual confirmation (aerial or ground monitoring in late spring/early summer), and vacated dens were visited in late summer or autumn–dens were the main source of pup scat. A few samples were collected opportunistically (n = 4); pack assignment was possible for one of these scats based on territory locational information (wolf ID: wolf8).

Scats were stored at -80°C for at least one week to deactivate the eggs [49], and then were partitioned into subsamples for different purposes (Fig 1): (1) wolf genotyping and sex identification, (2) parasite genotyping, and (3) endocrinology. For each sample, the tip of the scat was removed first for wolf DNA extraction, then the rest of the sample was homogenized as best as possible in the whirl-pack before partitioning for parasite genotyping and endocrinology. Our initial dataset was 207 scats. Scats that were misidentified as wolf (often coyote) or were of very poor quality (e.g., too dry, mostly hair, or mixed with snow during collection) were not analyzed, leaving a final sample size of 110 usable scats. All biohazard regulations were strictly followed during the collection, transport, partitioning, and shipping of scat samples. Individual host genotypes were compared to those resulting from cheek swabs, blood, tissue, and fecal swabs samples from wolves during live capture. Twenty-nine collared (or necropsied) wolves were genotyped, representing 81% of wolves present in the focal packs during sampling.

### Wolf genotyping and sex identification

Noninvasive DNA analysis was conducted at the USDA-APHIS-WS National Wildlife Research Center (NWRC), Fort Collins, Colorado, USA, following methods described by [27] (Fig 1A). Wildlife Genetics Laboratory personnel extracted DNA from scat samples in concordance with the protocol for DNA isolation using QIAamp Fast DNA Stool Mini Kits (Qiagen, Valencia, CA, USA). Tissue, saliva swab, and blood samples from captured wolves were used to isolate and purify DNA using a DNeasy Blood and Tissue Kit following protocols for QIAcube automation (Qiagen). Scat DNA extractions, polymerase chain reaction (PCR), and post-PCR processing were conducted in separate rooms. Each extraction and PCR was conducted with negative controls, and two individuals performed all genotypic scoring to ensure consistency.

Ten microsatellite loci were multiplexed in three panels to genotype wolves [27]. Each scat sample underwent a multiple tubes approach [50, 51] with two separate extractions and three PCR replicates each, totaling six replicates per scat. Samples were removed from further PCR and analysis if the first panel, consisting of four loci, did not produce a consensus call from at least two loci. We determined consensus genotypes from the six replicates following a conservative set of rules requiring two or more matching heterozygote alleles, three or more matching homozygote alleles, or other consensus-calling scenarios outlined in [51]. Samples with fewer than 7/10 loci successful amplification were removed from the dataset and not considered in further analyses. We then used the program gimlet [52] to quantify genotyping error rates. Sex was determined using the primers DBX6B and DBX6lv for X chromosome, and DBY7A and DBY7lv for Y chromosome [53]. Amplified products were then visualized on a QIAxcel Advanced (Qiagen) using a minimum of three replications per sample, and sex identification had to be confirmed by at least two replicates.

We compared known individual genotypes from the Yellowstone wolf population (i.e., collared wolves) with observed genotypes from fecal samples (Fig 1A). Wolves were considered to have the same genotype when at least 80% of alleles matched, allowing a maximum of two mismatching alleles to account for allelic dropout and false alleles. This allowed us to quantify the number of unique wolves sampled, detect repeatedly sampled individuals, and match scats with collared wolves. To detect genotype matches we used the Excel add-in GenAlEx 6.502 [54]. We allowed that zeros in the data could be possible matches to amplified alleles from another sample.

### Parasitology

Retrieval of parasite DNA from wolf scat and subsequent molecular analysis was carried out at the National Reference Laboratory for Parasites, Animal and Plant Health Agency (APHA), York, UK (Fig 1B). Parasite DNA was extracted from wolf scat samples (n = 104) using the QIAamp DNA Stool Mini Kit (Qiagen) according to the manufacturer’s instructions. A published multiplex PCR for the detection of cestode DNA in carnivore scat was used to amplify diagnostic fragments within the mitochondrial genome (Fig 1B); this assay can detect one taeniid egg which was quantitatively assessed to be equivalent to 7000 targets [20]. Cestodes of interest included *Echinococcus granulosus sensu lato*, *E*. *multilocularis*, *Taenia spp*., *Mesocestoides spp*., and *Dipylidium caninum*. Reference genomic DNA extracted from parasite tissues and previously verified by sequencing and PCR grade water were used as positive and negative controls, respectively. All amplified products were sequenced, and chromatograms were examined using FinchTV viewer (Geospiza, Seattle, Washington, USA). Nucleotide sequences were compared against the NCBI database using the Basic Local Alignment Search Tool (BLAST, U.S. National Library of Medicine).

A total of 104 samples were analyzed at APHA, six fewer than analyzed at APHIS, five of which failed to amplify at APHIS and were very dry, therefore we decided not to send them to APHA. One scat was overlooked during shipping to APHA, so instead, it was analyzed via a fecal float at Montana Department of Livestock Laboratory, Montana, USA (see S1 File).

### Endocrinology

Endocrinology was conducted at the St. Louis Zoo Endocrinology Lab, St. Louis, Missouri, USA (Fig 1C). Fecal steroids are solubilized using a previously published method [55]. Briefly, approximately 0.5 g of fecal material was shaken overnight in 5-ml phosphate-saline buffer containing 50% methanol, 0.1% bovine serum albumin, and 0.05% Tween 20 (polyoxyethylene sorbitan monolaurate, a surfactant). Following centrifugation at 4,000 g for 60 minutes, supernatants were decanted and stored in evaporation-proof vials at -80°C until assay. Fecal glucocorticoids were measured using a commercially available radioimmunoassay (DA I-125 Corticosterone RIA, ICN MP Biomedicals, Solon, OH, USA). Although cortisol is the primary circulating glucocorticoid of wolves, it is excreted in scat as a mixture of glucocorticoid metabolites. This assay was selected because it cross-reacts with fecal glucocorticoid metabolites in a variety of mammalian species [56]. The detection limits of the assay were ~0.26 to 20.0 ng/ml. Assays were performed according to the manufacturer’s instructions, with the exception that standard diluent was added to the fecal extracts, and fecal extraction buffer (containing 50% methanol) was added to the standards. Standards, samples, and quality controls were assayed in duplicate for all assays. Hormone concentrations were determined as ng/ml and then divided by the dry weight of the extracted scat to give results as ng/g scat.

### Serology

Blood serum samples were obtained during wolf live capture. Sera was screened for antibodies to canine distemper virus, *Toxoplasma gondii*, and *Neospora caninum* at the Cornell Animal Health Diagnostic Center (Ithaca, NY, USA). See S1 File for specific assays performed and titer levels considered seropositive/seronegative.

### Statistical analyses

We constructed two types of generalized linear mixed models: one model predicting cestode infection based on wolf biological and social characteristics that we suspected would influence infection (‘cestode model’); the second model examined how environmental conditions influence the success of host genotype success (‘success model’).

### Cestode model

We were interested in a suite of variables that we predicted could explain variation in cestode infection (S1 Table in S1 File), including AGE (pup/adult), SEX (female/male), SEASON (summer/winter), STRESS (fecal glucocorticoid measurement; log-transformed to meet the normality assumption), PACK SIZE (varies summer/winter, scaled and centered), and wolf population DENSITY (varies summer/winter, scaled and centered). We were also interested in diet as these parasites require intermediate hosts, but the prey carcass site method used to record diet was not sensitive enough to parse the all prey consumed, and packs primarily consumed elk. Instead, PACK SIZE can be interpreted as a proxy for diversity in diet because larger packs are able to kill larger prey [57], therefore we predict that smaller packs have more diverse diets, and consequently, greater parasite exposure. We estimated wolf age class (pup ≤1 year old or adult >1 years old) using scat diameter and ages of known wolves, with the goal of minimizing the amount of error in age class classification (S1 File). Using a 2.1 cm cutoff (pup scat diameter ≤2.1 cm, adult scat diameter >2.1 cm) correctly classified all known-aged wolves except one scat from a collared pup, however, her other two scats were within the pup classification. This cutoff resulted in the 34 genotyped wolves being classified as 22 adults and 12 pups. We also ran the cestode model using just collared wolves, which allowed us to use a finer age resolution (numeric age instead of age class) and add the following variables of interest: coat COLOR (black/gray), breeding status (BREEDER 0/1), and exposure to other parasites (positive/negative) (S1 Table in S1 File). Pairwise comparisons were done with Fisher’s Exact Test for categorical variables. We expected that wolves would be more likely to test positive for an infection with higher stress, smaller packs, with greater population density, in males, and as wolves age (i.e., adults > pups). Using the collared subset, we expected breeders and gray-colored wolves would be more likely to test positive for an infection due to documented higher stress levels and potential interactions with immune response [58, 59]. We intended to include seroprevalence data for two parasites and one virus that could weaken host immune responses: *Neospora caninum* (NEO), *Toxoplasma gondii* (TOXO), and canine distemper virus (CDV), but these data were incomplete. Instead, we report serological prevalence and conduct Fisher’s Exact Tests comparing seroprevalence among uninfected wolves and wolves infected with cestodes.

All variables in the cestode model were screened for collinearity prior to model implementation (*Spearman’s r* < 0.6). We modeled the probability that a wolf tested positive for an infection using a binomial error distribution and a logit link. For the cestode model, random effects on the intercept were initially included to account for variation in infection; these included a categorical pack membership variable (PACK) and unique wolf ID (WOLF). However, models would not converge when both random effects were included simultaneously (singularity), therefore only WOLF was retained.

### Success model

Weather variables used in the success model were collected from a weather station in Tower Falls, Wyoming, which was centrally located in our study area (National Interagency Fire Center Remote Automated Weather Station: latitude 44.9169, longitude -110.420556). The duration of the scat, or days from deposit to collection, was estimated for most usable samples (98%). Scat deposit day was estimated as the day the GPS cluster was created for scats collected at GPS clusters, the date of prey death for scats collected at prey carcass sites, or the median day between the first GPS fix and collection for scats collected at den sites. We calculated exposure durations as the number of days between the scat deposit time and noon on the day of collection rounded to the nearest whole day. Although other weather variables have been suggested to affect DNA quality, such as relative humidity, we parsed the weather variables to mean high temperature (HIGH TEMP in degrees Fahrenheit, scaled and centered), total precipitation (PRECIP in inches, scaled and centered), total days elapsed (DAYS), PERIOD (summer, winter, or denning), and COVER as open (0) or closed (1) canopy because they have ample support in the literature [17, 25, 51, 60–62].

Only scats with complete metadata were used in the success model (removed n = 3 samples). All variables were screened for collinearity prior to model implementation (*Spearman’s r* < 0.7); DAYS was correlated with PRECIP and was run in a separate univariate analysis. In addition, DAYS was log-transformed to meet the normality assumption. We modeled the probability that a wolf scat sample was successfully genotyped using a binomial error distribution and a logit link. We considered a sample to be successfully genotyped when it met our criteria of 7/10 amplified loci using the six total replicates. SITE was included as a random effect on the intercept to account for scats collected at the same location.

All model construction, analysis, and visualization was performed in R [63], using the packages lme4 [64], ggplot2 [65], dplyr [66], arm [67], pscl [68], cowplot [69], and pwr [70].

## Results

### Dataset and laboratory results

Of the 110 samples across four packs, we collected an average of 27.5 samples per pack and 36.7 samples per season. We successfully genotyped 49/110 fecal samples (45% success rate). Of the 49 genotypes, we detected 34 unique genotypes: 23 wolves were sampled once, seven wolves were sampled twice, and four wolves were sampled three times. Eleven collared wolves were detected and 23 unique uncollared wolves were detected. Sexes were sampled nearly equally: 18 females and 16 males; one scat sample was identified as male but matched with a known female, thus we considered it to be the known female (1049F, 19/20 alleles). Allelic dropout rates and rate of false alleles were low at 0.143 and 0.021, respectively, across replicates per individual scat. Median glucocorticoid measurements of unique wolves ranged from 16.2 to 694.4 ng/g (median = 62.2 ng/g)–see S1 File for additional glucocorticoid results and analyses.

Three cestode species were detected using all samples, and at least one cestode species was detected in 29 out of 104 samples (27.9%): 21.2% *E*. *granulosus sensu lato* (n = 22, of these, 17 were identified as *E*. *canadensis* and five could not be sequenced), 1.9% *E*. *multilocularis* (n = 2), 3.8% *Taenia serialis* (n = 4), and one scat was infected with an unidentified *Taenia spp*., *Mesocestoides spp*., or *Dipylidium caninum* (Table 1). There were no co-infections within the same sample, but one wolf tested positive for two different species at two sampling events (1005F), and was therefore only counted once in the apparent prevalence estimate. Prevalence in the 34 unique wolves was 32.4% *E*. *granulosus sensu lato* (n = 11, 8 of which were identified as *E*. *canadensis*), 2.9% *E*. *multilocularis* (n = 1), and 5.9% *Taenia serialis* (n = 2), totaling 38.2% (13/34) cestode prevalence (Table 1). Three wolves became infected at subsequent sampling events: two wolves became infected with *T*. *serialis* (1005F, wolf 7), and one became infected with *E*. *multilocularis* (wolf 8). 1005F was the only wolf where a previous infection was not detected in later sampling (*E*. *granulosus sensu lato*). While we suspect all wolves with detected *E*. *granulosus sensu lato* are infected with *E*. *canadensis* (following [30, 71, 72]), we refer to these infections as *E*. *granulosus sensu lato* when reporting fecal and apparent prevalence due to the five samples that could not be sequenced.

**Table 1:** Fecal prevalence (number of detected infections/number of samples) and apparent maximum prevalence (number of infected wolves/number of unique wolves) in northern Yellowstone wolves years 2018–2020.

Cestode	Fecal prevalence (number of detected infections/number of samples)	Apparent maximum prevalence (number of infected wolves/number of unique wolves)
*E*. *granulosus sensu lato*	4.8% (5/104)	8.8% (3/34)
*E*. *canadensis*	16.3% (17/104)	23.5% (8/34)
*E*. *multilocularis*	1.9% (2/104)	2.9% (1/34)
*T*. *serialis*	3.8% (4/104)	5.9% (2/34)
*Taenia spp*., *Mesocestoides spp*., or *Dipylidium caninum*	0.96% (1/104)	0.0% (0/34)
**Overall**	**27.9% (29/104)**	**38.2% (13/34)** *

*One wolf (1005F) was infected with different cestodes on two different sampling occasions (*E*. *granulosus sensu lato*, *T*. *serialis*), and therefore was only counted in apparent maximum prevalence once–thus overall prevalence is 13/34 although the rows add to 14.

### Statistical analysis–cestode model

**Fig 2:**
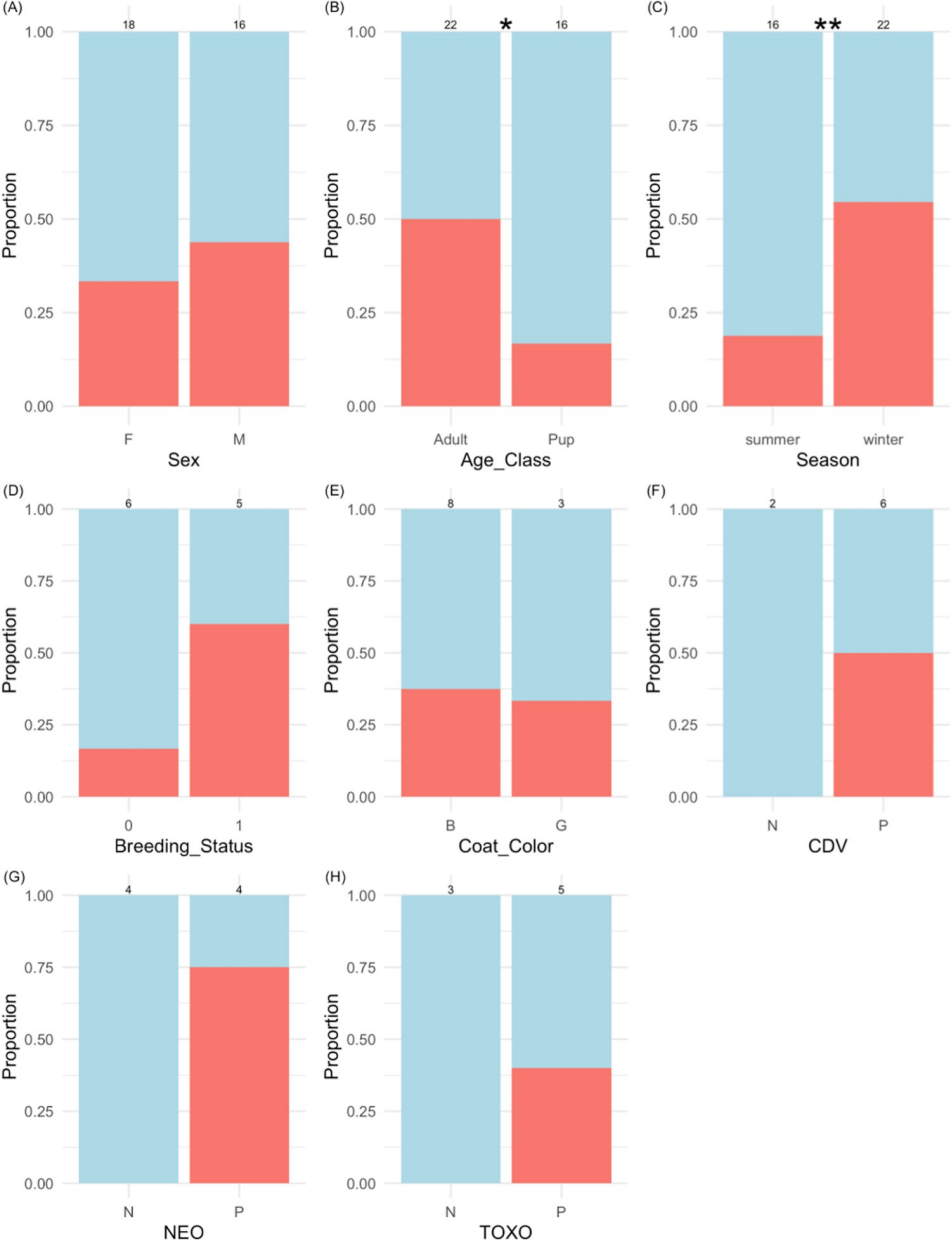
Proportion of cestode-infected (red) and uninfected (blue) wolves stratified by **(A)** sex (Female/Male), **(B)** age class, **(C)** season, **(D)** breeding status (0 = non-breeder, 1 = breeder), **(E)** coat color (Black/Gray), **(F)** canine distemper virus exposure (Negative/Positive), **(G)** *N*. *caninum* infection (Negative/Positive), and **(H)** *T*. *gondii* infection (Negative/Positive). Stars * and ** denote *p* < 0.10 and *p*<0.05 using Fisher’s Exact Test; sample sizes are displayed above columns.

**Fig 3:**
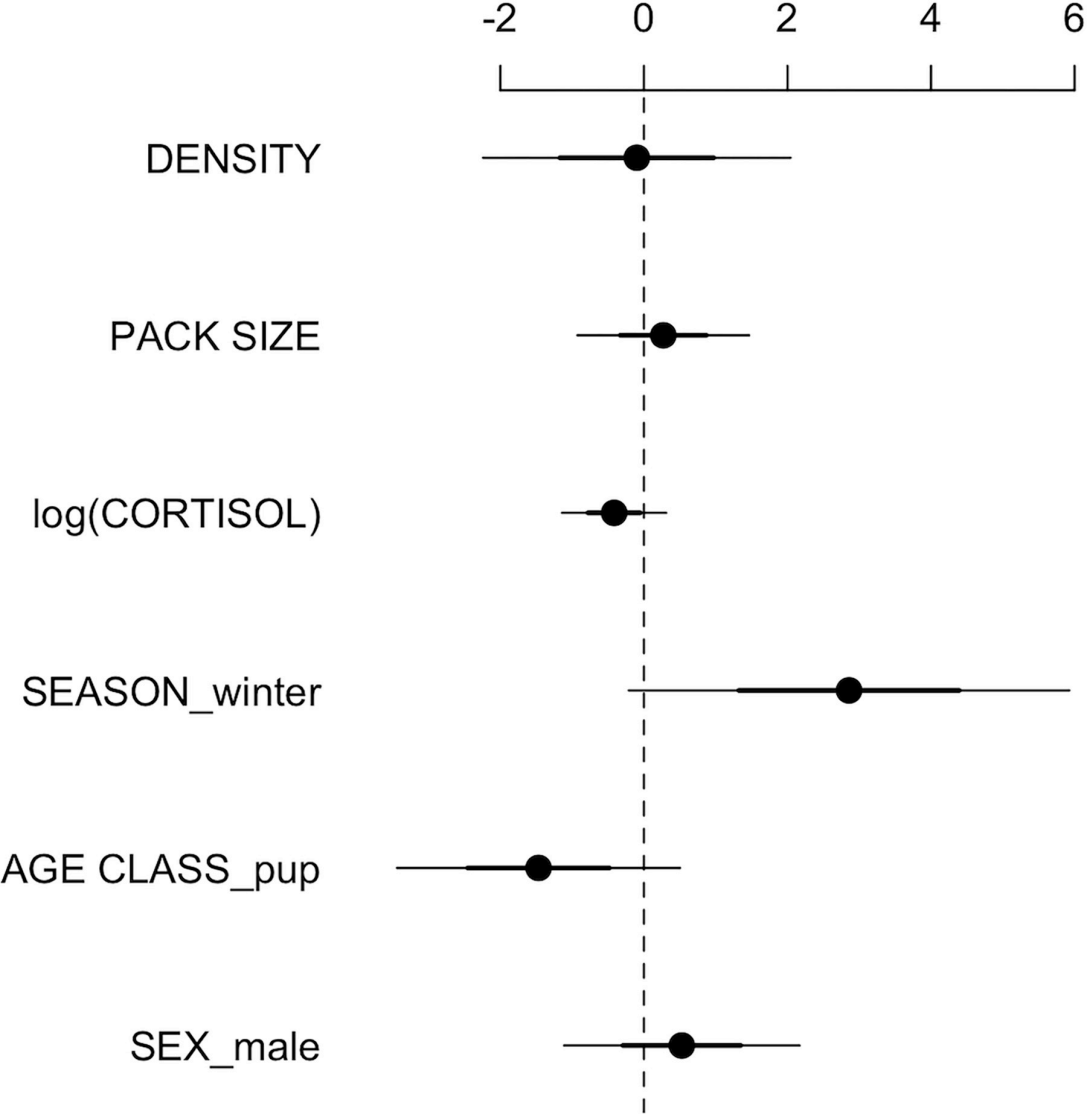
Coefficient estimates for the cestode model (log-odds ratios; points). Error lines represent 50% (thick) and 95% (thin) confidence intervals. For categorical variables, SEASON winter is with reference to summer, AGE CLASS pup to adult, and SEX male to female.

Wolves sampled during the winter were more likely to test positive for cestode infection (odds ratio = 5.0, *p* = 0.04); the proportion of infected male wolves was slightly greater than that of females (odds ratio = 1.5, *p* = 0.39), and adults greater than pups (odds ratio = 4.8, *p* = 0.06), but there was no statistical difference (Fig 2). However, power for detecting large effects (≥0.35, α = 0.05) was only 0.26, thus we were unlikely to detect statistically significant effects in these analyses. Pairwise analysis results were supported in the cestode model with relatively large effect sizes, but not statistical significance (SEX (MALE): β = 0.64, *p* = 0.55; AGE CLASS (PUP): β = -1.45, *p* = 0.25; SEASON (WINTER): β = 3.09, *p* = 0.11; Fig 3). DENSITY (β = -0.03, *p* = 0.98), PACK SIZE (β = 0.37, *p* = 0.64), and log(CORTISOL) (β = -0.43, *p* = 0.35) had negligible effects on the probability a wolf had a cestode infection (Fig 3). The cestode model accounted for 31% of the variation in infection status (pseudo-R^2^), and wolf random effects were moderate (variance = 2.2).

In the cestode model using only collared wolves, older wolves (numeric AGE β = 11.56, *p* = 0.06) and breeders (BREEDER β = 11.73, *p* = 0.18) tended to be more likely to test positive for a cestode infection, and gray-colored wolves tended to be less likely to test positive (COLOR β = -38.1, *p* = 0.09), but no variables were significant at an alpha-level of 0.05. The cestode model accounted for 39% of the variation in collared wolf infection status (pseudo-R^2^). Similarly, proportion of infected wolves stratified by breeding (odds ratio = 6.1, *p* = 0.20) and coat color (gray odds ratio = 1.2, *p* = 0.79), and infection with canine distemper virus (*p* = 0.36), *N*. *caninum* (*p* = 0.07), and *T*. *gondii* (*p* = 0.36) were not significant using Fisher’s Exact Test. Power was weak in these analyses due to small sample size, especially for serological exposure (i.e., poor estimation of odds ratios).

### Statistical analysis–success model

**Fig 4:**
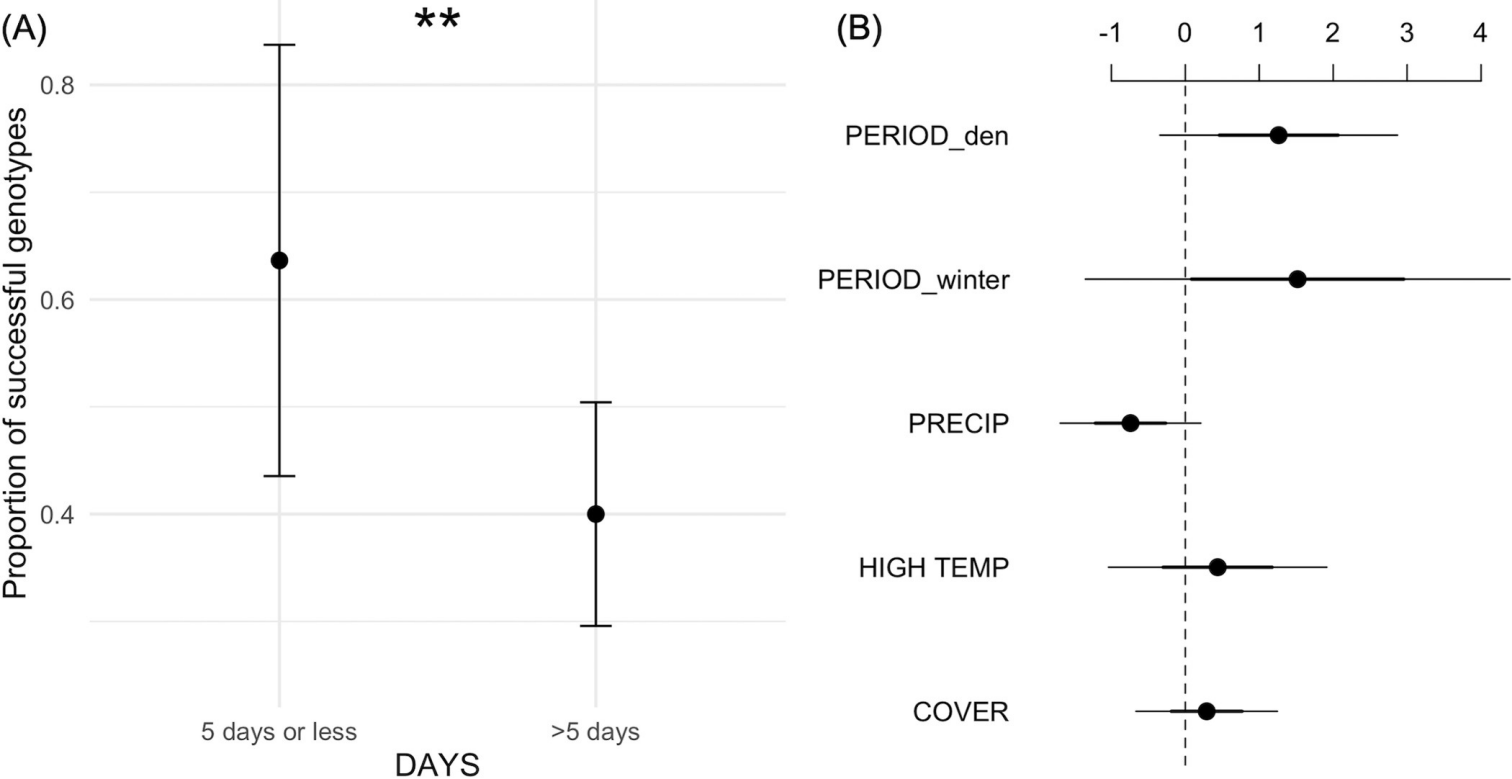
**(A)** Proportion of scats successfully genotyped when collected within 5 days of deposition (n = 22), or more than 5 days (n = 85), with 95% confidence intervals. Stars ** denote *p* < 0.05 using Fisher’s Exact Test. **(B)** Coefficient estimates for the success model (log-odds ratios; points). Error lines represent 50% (thick) and 95% (thin) confidence intervals. Collection PERIOD den and winter are with reference to summer, and COVER is closed with reference to open canopy.

The probability that a scat sample was successfully genotyped declined as the number of days elapsed between deposit to collection (log(DAYS): β = -0.43). Although this effect did not reach statistical significance in the GLMM (*p* = 0.09), time elapsed had a substantial compounding effect; for example, if more than five days elapsed from deposit to collection, the probability that a scat sample was successfully genotyped dropped by approximately 24% on average (Fig 4A; Fisher’s Exact Test odds ratio = 2.6, *p* = 0.04).

The probability that a scat sample was successfully genotyped tended to decline with increasing precipitation (PRECIP: β = -0.75, *p* = 0.13; Fig 4B). Scat samples were more likely to be successfully genotyped when collected during the winter (β = 1.50, *p* = 0.30) and denning (β = 1.27, *p* = 0.12) compared to summer. PRECIP and PERIOD had large effect sizes but were not statistically significant predictors of success. Exposure to radiation (COVER = 1: β = 0.28, *p* = 0.58) and temperature (HIGH TEMP: β = 0.42, *p* = 0.57; Fig 4B) had no impact on the probability of success. Sampling site accounted for a small amount of variation in genotyping probability (variance = 0.03).

## Discussion

Helminth infections are pervasive among wildlife species and can reduce host health and fitness, yet these parasites are generally understudied in large carnivores due to logistical and ethical constraints. Here we combined multiple data streams to identify the cestodes infecting a wide-ranging carnivore host, as well to identify the host characteristics that influence these parasite infections. This approach allowed us to estimate apparent cestode prevalence using known wolves (38%), which was 10% greater than fecal prevalence (28%). *Echinococcus granulosus sensu lato* was the most commonly identified cestode, while *E*. *multilocularis* and other *Taenia spp*. were rare. At least two wolves (four scat samples) were infected with *Taenia serialis*–to our knowledge, this is the first time *T*. *serialis* has been detected in North American wolves south of the Yukon and Northwest Territories, or in North America within the last 50 years [73]. Adult wolves were more likely to have a cestode infection than pups, as were wolves sampled in the winter compared to summer. Wolf genotyping success declined in the summer, with increasing precipitation, and as more time elapsed between scat deposit and collection. While this project demonstrates the potential for noninvasive parasitology and disease ecology research, limitations included small sample sizes and degraded scat samples for molecular analysis.

We were able to match wolf scat DNA to known individuals, either collared or uncollared (Fig 1), providing the opportunity to better understand the biological and social characteristics associated with cestode infection. Previous wolf parasitology research in Yellowstone National Park relied on observing wolves defecating and collecting samples quickly after [15, 74], but this approach is not possible for many wildlife species, including wolves in much of their range. Others conducting wolf parasitology research also identified unique wolves via genotyping [22], but low genotyping success resulted in few wolves being identified, and they lacked corresponding individual-level covariates. In Yellowstone National Park, wolves are monitored year-round including records of breeding status, age, pack size, population density, coat color, and exposure to parasites via serology (e.g., canine distemper virus, *N*. *caninum*, and *T*. *gondii*). As predicted and corresponding with other gastrointestinal parasitology studies [75], wolves were more likely to be infected as they aged due to a longer exposure period. Contrary to our predictions, estimates for the effects of pack size and population density were near zero (Fig 3), and coat color was not associated with infections (Fig 2E). The proportion of infected breeders was greater than that of nonbreeders, but small sample sizes presumably contributed to lack of statistical significance. In terms of co-infections, the proportion of cestode-infected wolves exposed to canine distemper virus, *N*. *caninum*, and *T*. *gondii* was greater than wolves not exposed to these infectious agents, yet small sample sizes prevented statistical evaluation of these co-infections (Fig 2F–2H). Helminth and other infectious pathogen co-infections may be an intriguing future area of research as these co-infections can incur immunological tradeoffs within a host. For example, immune suppression caused by nematode infections promotes subsequent bovine tuberculosis infection in African buffalo (*Syncerus caffer*; [76]).

Wolves are highly social species that live in packs with dominance hierarchies where there is one primary breeding pair [77]. In social species, dominant individuals can have elevated cortisol associated with the social and physical stress of dominance [58, 78, 79]; previous research on Yellowstone wolves has supported this relationship between breeding and stress [74], but others have not [80]. Yet research on other social species, like wild olive baboons (*Papio anubis*) and spotted hyenas (*Crocuta crocuta*), demonstrated that subordinate individuals can experience higher stress than dominant individuals [81, 82]. In this study, non-breeders tended to have lower stress than breeders (S5B Fig in S1 File; non-breeders: range 55.2–379.1, median 80.0; breeders: range 16.5–260.0, median 26.6; units ng/g of scat) but this was not statistically significant (one-tailed t-test: t = -0.77, *p* = 0.23). In addition, increased stress has been linked to parasite infections in wildlife hosts in terms of higher infection intensity [83] and probability of infection [84]. Here, cortisol was not an important predictor of cestode infection.

We identified den sites as a source of relatively higher scat quality for molecular analyses, and because it is logistically easier to collect scats from dens than at GPS clusters year-round, this is a promising source of host and parasite samples. Analysis of scats collected at den sites have been used to estimate population abundance and pack relatedness [85, 86], and analysis for parasites could readily be added to these projects. However, no infected pup scats were collected at dens in our sample, and wolves were more likely to be infected as they aged–two pups tested positive for infection at around 8 months old. Additionally, results from the cestode model suggest that wolves were less likely to test positive for infections in the summer, which overlaps with the wolf denning period. Therefore, samples collected at dens are most useful for examining infections in wolves at least one year old, but might underestimate population prevalence.

Gray wolves and other canids are the definitive host for the cestodes identified, and specifically, *E*. *canadensis* is likely endemic in wolves in western North America [15, 30, 31, 71, 72]. Therefore, detection of this parasite also provides information about parasite infections in sympatric prey species, as well as the prey consumed by wolves in Yellowstone [32]. Information about *E*. *granulosus sensu lato* infections in intermediate hosts in the Yellowstone region is scant, although it has been detected in mountain goats (*Oreamnos americanus*) [71]. The *Echinococcus* strain we detected most commonly in this study (*E*. *canadensis*) is associated with cervid intermediate hosts [87–89], and because elk are the primary prey species of Yellowstone wolves [90], it is probable they are also the primary intermediate host. The intermediate hosts for *T*. *serialis* are rabbits and hares (but see [91] who identified a novel cervid intermediate host), yet these hosts are known to be uncommon [92] and are rarely detected using carcass-search methods employed in Yellowstone National Park as wolves will consume the entire carcass [93]. This could explain the low number of detections of *T*. *serialis* in this study. We were unable to address the effects of diet on parasite infection here, but our findings may be useful as preliminary research for future studies, such as using isotopes to identify small prey species in wolf diets [94], or analyzing parasites in large sympatric prey species.

### Evaluating this methodology for future work

We briefly evaluate the utility of this combination of methods for future wildlife host or parasite research, and make suggestions from our experiences. First, depending on the study species and system, field work can be extensive and challenging. If possible, we suggest employing our methods in a system that already has research programs in place. Although our collection methods were noninvasive, our project benefited from long-term research in many aspects, including serum sample collection and host variable analysis (e.g., age determined during wolf captures).

Second, molecular laboratory work is costly, which may prevent some from being able to conduct this work. More specifically, the cost per sample could range from $200-$300 for laboratory analyses including parasitology, endocrinology, wolf genotyping, and sex identification, and additionally, there are the costs of field technician salary (hourly rate, approximately 8 hours per day for 80+ days for two technicians), shipping, and supplies. Due to the high resolution of our statistical analyses (e.g., individual-level), and considering the moderate success rate of wolf genotyping, only about half of our samples could be analyzed. Importantly, however, the costs and logistical challenges of large live-animal capture and full necropsies exceed the cost per sample using our methods, and therefore our methods could provide a suitable alternative in some settings.

Third, research using fecal DNA should focus on maximizing the success of laboratory analyses, such as reducing the amount of time between deposition and collection as much as possible and collecting a large number of samples [95]. This was not possible in the national park setting where we conducted this project, and thus scats were collected, on average, 17 days following deposit (range 1–75 days). Despite this, we had moderate genotyping success at 45% (49/110). In agreement with other studies, we found that DNA quality was better retained in the winter, at dens, when scats are protected from solar radiation, and when scats are collected with time minimized between deposition and collection in the field [17, 95, 96]. The climate in Yellowstone National Park and other northern temperate regions is therefore moderately favorable year-round and ideal in the winter for noninvasive molecular research, suggesting that samples must be collected sooner after deposition in wetter, warmer climates to achieve similar genotyping success. Without the sampling design limitations of our project [95], these methods could be a powerful approach for assessing noninvasive parasite infection patterns in a population of terrestrial mammals.

Fourth, our study built on and improved previous research by applying PCR-based parasite detection techniques, as well as identifying the unique wolves sampled and matching them with their sample. A significant benefit of molecular diagnostics is the ability to detect fragments of oocysts, whose shedding is heterogeneous, as well as cestode fragments, thereby increasing the confidence of parasite detections [97, 98]. By identifying unique wolves and matching them to their scat samples, we ensured we were not unknowingly including repeatedly sampled individuals. The apparent prevalence estimate using one sample from each unique wolf was 10% greater than the fecal prevalence estimate (38% vs 28%, respectively). Still, PCR detection methods may be imperfect, and evaluation of diagnostic test features such as sensitivity and specificity are critical for interpreting results, especially across studies and differing detection methods [99].

## Conclusion

We identified cestode infections in a wide-ranging carnivore using a combination of noninvasive methodologies. We demonstrate the difference between prevalence estimates using pooled fecal samples and using samples from unique individuals. In Yellowstone wolves, *Echinococcus canadensis* was the most commonly identified cestode infection, and infections were influenced by host sex and season of sampling. We did not detect associations with other host characteristics such as coat color, sex, or breeding status at least partially due to small sample sizes. Despite potential limitations and considerations, our methods are a promising alternative to collecting samples during live-captures or necropsy but would have benefitted from sampling design that focused on ideal DNA sampling in the scat collection design.

## Supporting information

S1 File This document contains five sections: (I) Additional information about parasitology and serology, (II) Scat diameter and aging, (III) Cortisol data and analysis, (IV) Model covariates considered, and (V) Reference.
(DOCX)
