# Supplementary material for: Evaluating noninvasive methods for estimating cestode prevalence in a wild carnivore population
Source: PLoS One. 2022 Nov 15;17(11):e0277420. doi: 10.1371/journal.pone.0277420 (PMC9665365; doi:10.1371/journal.pone.0277420)
Supplement: S1 File — This document contains five sections: (I) Additional information about parasitology and serology, (II) Scat diameter and aging, (III) Cortisol data and analysis, (IV) Model covariates considered, and (V) Reference. (DOCX) [file pone.0277420.s001.docx]

**Supplementary Material for**

**Evaluating noninvasive methods for estimating cestode prevalence in a wild carnivore population**

Ellen E. Brandell, Madeline K. Jackson, Paul C. Cross, Antoinette J. Piaggio, Daniel R. Taylor,

Douglas W. Smith, Belgees Boufana, Daniel R. Stahler, Peter J. Hudson

SECTIONS:

| I | Additional information about parasitology and serology |
| --- | --- |
| II | Scat diameter and aging |
| III | Cortisol data and analyses |
| IV | Model covariates considered |
| V | References |

**SECTION I**

All wolf sera samples were analyzed at Cornell Animal Health Diagnostic Center, Ithaca, New York, USA. A virus/serum neutralization assay was performed for canine distemper virus antibody detection where positive was considered ≥1:16 dilution. An indirect fluorescent assay was performed for *N. caninum* antibody detection, which provides a positive/negative result. An ELISA or Modified Agglutination Test was performed for *T. gondii* antibody detection, which provides a positive/negative result or titer value, respectively; *T. gondii* positive was considered ≥1:25 dilution. Wolves were captured in winter, typically December through March, thus serological sampling could occur prior to scat sampling in a year (i.e., January or February) or following scat sampling that year (i.e., December). For wolves sampled following scat collection, approximately five months passed from deposition to serum sampling.

A wolf was classified into one of four exposure statuses: positive, negative, unknown, or N/A. When a wolf tested positive prior to scat deposition or positive during the year of scat deposition, it was considered “positive.” A wolf was “negative” when it tested negative during the year of scat deposition. “Unknown” results occurred when a wolf had no detected exposure prior to scat sampling and was not sampled during the year of scat deposition (n=3 adults with partially or fully unknown exposure). Finally, N/A occurred when no assay was performed (n=1 pup).

One scat sample (scatID 18) was overlooked during the shipping procedure from Yellowstone National Park to the parasitology lab at the National Reference Laboratory for Parasites, Animal and Plant Health Agency, York, UK. Therefore, it was analyzed for cestodes at the [Montana Veterinary Diagnostic Laboratory](http://liv.mt.gov/diagnostic-lab/) in Bozeman, Montana, USA, using a fecal flotation method. For this, 3.0-5.0 g of feces was placed in a 15-ml conical tube and the remainder of the tube was filled with 12-14 ml of zinc sulfate (ZnSO_4_). The tube was centrifuged 1,200 rpm for 5 minutes. The tube was then placed in a tube rack and topped off with enough ZnSO_4_ to form a convex meniscus. A cover slip was placed on top of the tube for 10 minutes. Afterwards, the coverslip was positioned on a slide and examined for parasites on a microscope at 10x magnification. Intestinal parasites were identified based on egg/oocyst morphology. No parasites were detected in this sample.

**SECTION II**

We used wolf scat diameters to classify wolves into pup and adult age categories. A range of methods have been used to classify wolves of unknown age into adult and pup categories. For example, a 2.5-cm diameter cutoff [(Ausband et al. 2010; Stenglein et al. 2010)](https://paperpile.com/c/M6UgTW/WlSPM+s2MmD) was based on a comparison between wolf pup and coyote scat sizes [(Weaver and Fritts 1979)](https://paperpile.com/c/M6UgTW/J3HRk); a 1.8-cm diameter cutoff was used because it was one standard deviation less than the total mean scat diameter [(Trejo 2012)](https://paperpile.com/c/M6UgTW/pqPY7). Given that we were able to match collared wolves with their scats, we leveraged known ages of collared wolves to assign an informed cutoff between these categories.

There were 11 sampled collared wolves: 3 males (5 male samples), 8 females (12 female samples), 3 pups (all female, 5 pup samples), and 8 adults (12 adult samples) (Fig. S1). Adults and pups had distinctly different scat size distributions (Fig. S1A), and adult females and males had similar median diameters but males had much greater variation (Fig. S1B).


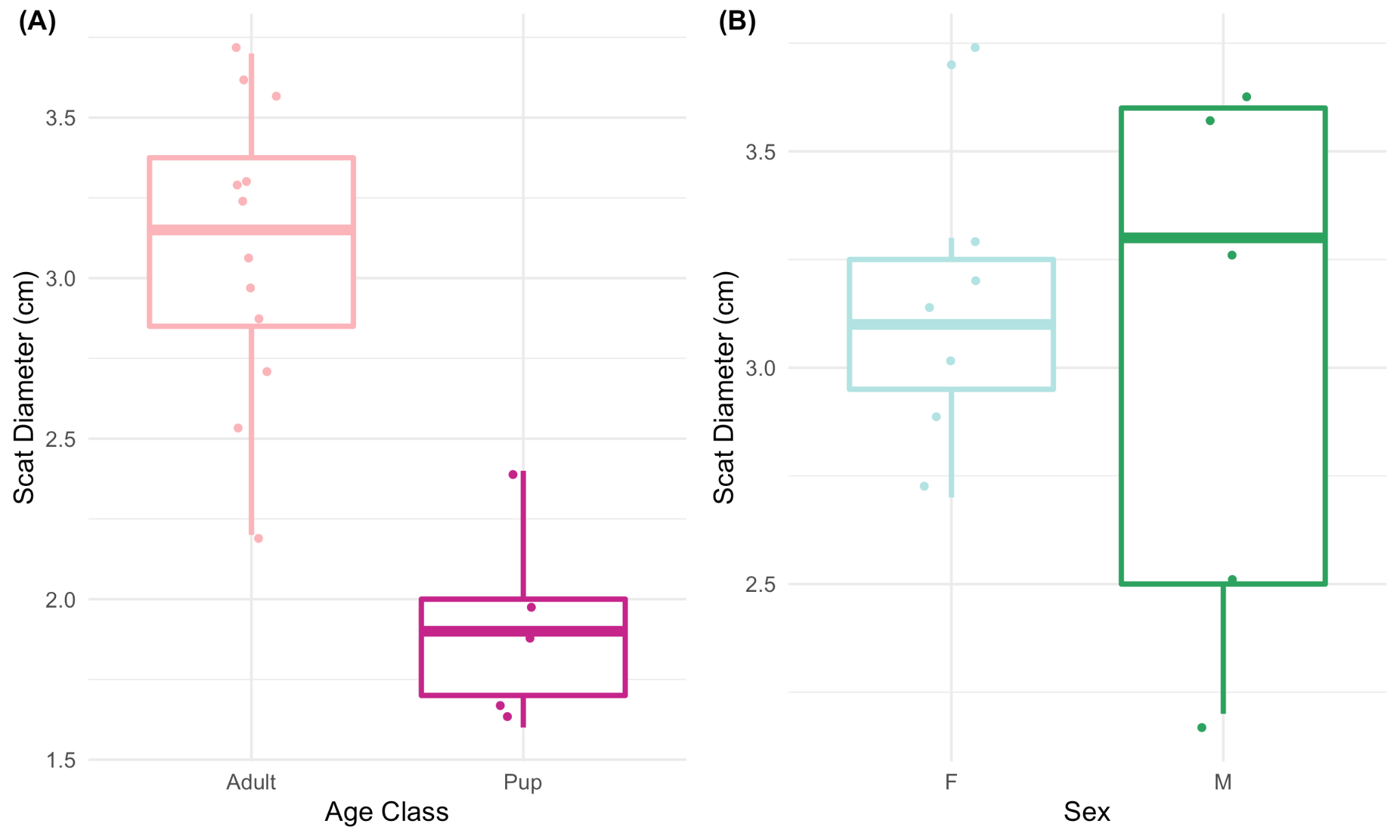


Figure S1. Scat diameter (cm) from collared wolves (n=11 wolves, n=17 scat measurements), grouped by (A) age class and (B) sex (only adult wolves; all sampled pups were female).

The largest known pup scat was 2.4 cm and the smallest adult scat was 2.2 cm, therefore no cutoff could perfectly classify wolves into the two age categories. 75% of pup scats were ≤2 cm in diameter (only 1 scat exceeded 2 cm); therefore we used this as a starting point for classifying all genotyped wolves (n=34 wolves, n=49 samples).

We iteratively increased the cutoff between wolf pups and adults from 2.0 to 2.5 cm in 0.1 increments and calculated the number of pups, the number of adults, and the number of wolves spanning both age classes (only possible with resampled wolves). For repeatedly sampled uncollared wolves, we followed this procedure with an assessment of the plausibility that a given wolf was a pup or an adult based on their collective samples. For example, wolf17 was sampled three times with scat diameters: 2.2, 3.1, and 2.5 cm. Although this wolf could be classified as a pup when using lower cutoffs, it tended to have scats larger than the majority of pups and was therefore classified as an adult.

Results from our iterative classification procedure indicate that lower cutoffs (2.0 and 2.1 cm) more accurately classified wolves into pups and adults than cutoffs ≥2.2 cm diameters. Age class mismatch was only indicated for one known wolf for cutoffs 2.0 and 2.1 cm, whereas cutoffs 2.2, 2.3, and 2.5 cm had three mismatches and 2.4 cm had two mismatches. In addition, the only mismatch for the lower cutoffs was a known pup (1228F), allowing us to correctly classify her. The total number of pups and adults differed between the 2.0 cm and the 2.1 cm cutoff: by increasing the cutoff, two wolves were classified as pups instead of adults (wolf10, wolf6), both with a scat diameter of 2.1 cm (2.0 cm: adults n=24, pups n=10; 2.1 cm: adults n=22, pups n=12). These scats were collected at kill sites in November and December and therefore could plausibly be adults or 7-8 month old pups, lending no additional information. Therefore, we selected the 2.1 cm cutoff to designate wolves as pups or adults (Fig. S2).


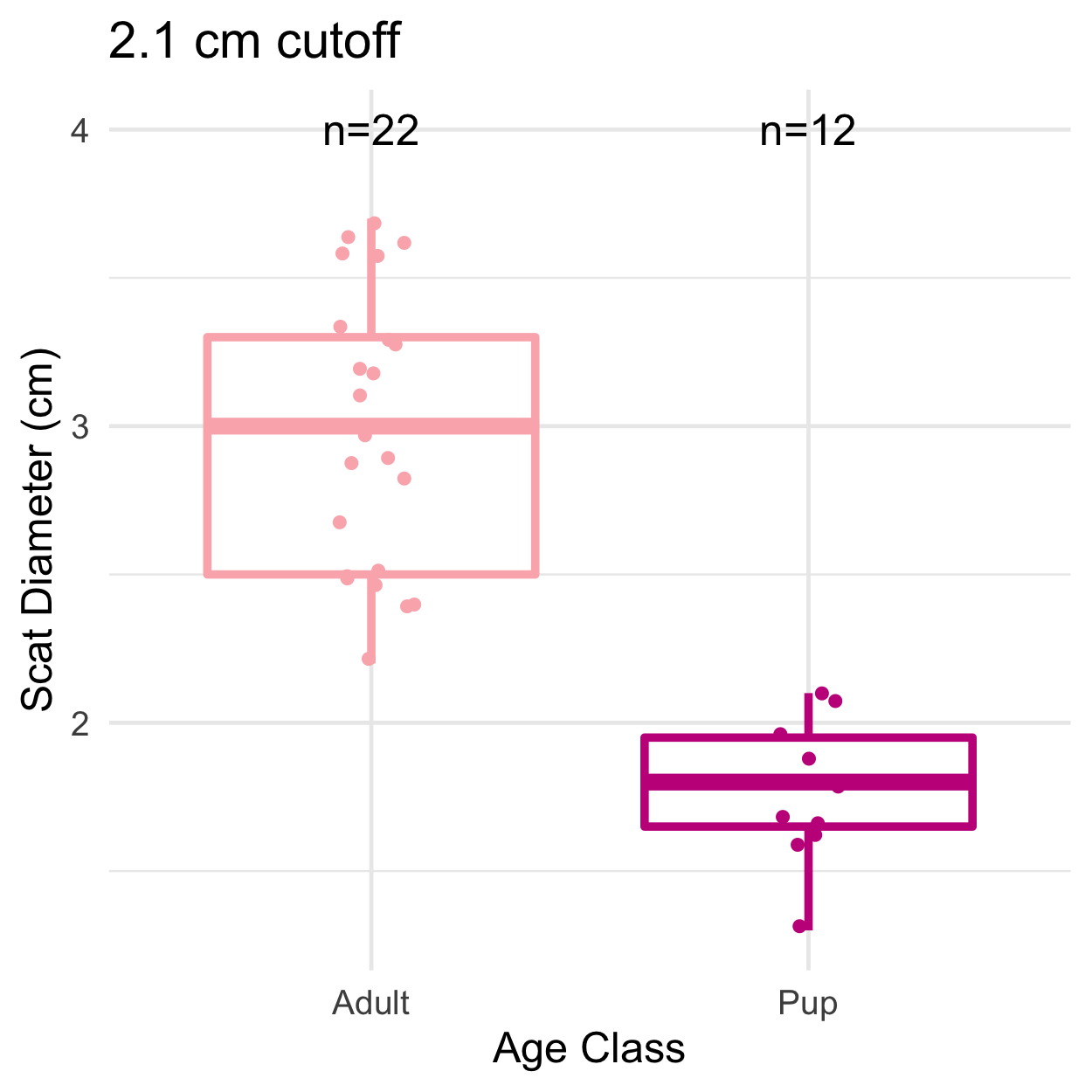


Figure S2. Scat diameter (cm) from genotyped wolves (n=34 wolves, n=49 scat measurements) using the selected 2.1 cm cutoff for classifying wolves as pups or adults.

We acknowledge there is some uncertainty in designating an age class based on a single scat sample from individual wolves, but we are confident that the vast majority of wolves are classified properly based on our assessment and use of collared wolf data.

**SECTION III**

There was a large range of cortisol (i.e., fecal glucocorticoid) measurements: 15.0-836.8 ng/g of scat, with a mean and median of 142.4 and 68.1 ng/g of scat, respectively (Fig. S3). Across genotyped wolves, cortisol did not differ by age class (Fig. S4A), sex (Fig. S4B) – although females had greater variation in cortisol measurements than males – or pack (Fig. S4C). For collared wolves, there were no significant differences in cortisol by coat color (Fig. S5A) or breeding status (Fig. S5B; non-breeders: range 55.2-379.1, median 80.0; breeders: range 16.5-260.0, median 26.6; one-tailed t-test: t = -0.77, *p* = 0.23). We used median cortisol measurements for repeatedly sampled wolves so each wolf has one data point in Figures S4-S5.


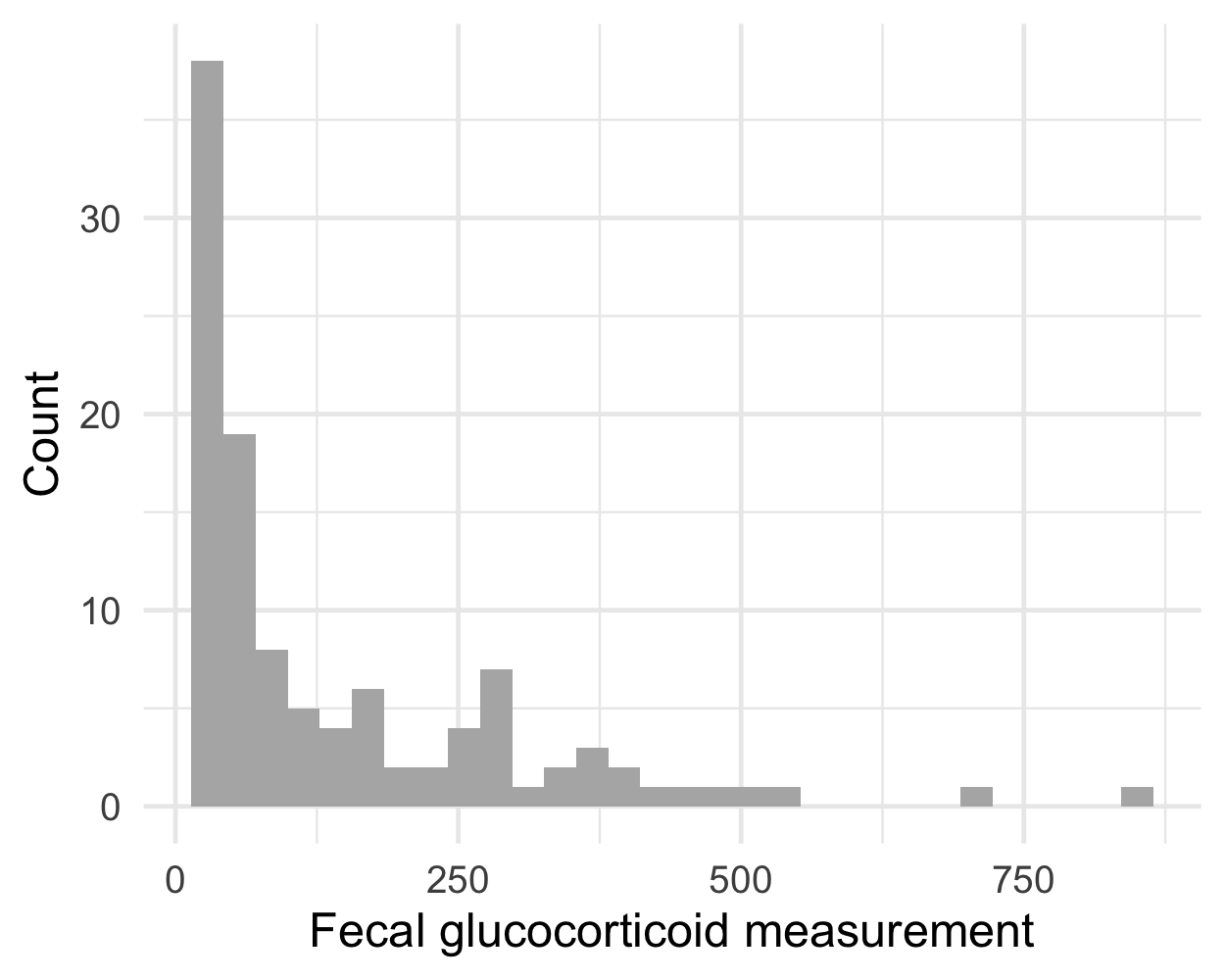


Figure S3. Histogram of all fecal glucocorticoid measurements (n=110, units=ng/g of scat).

**
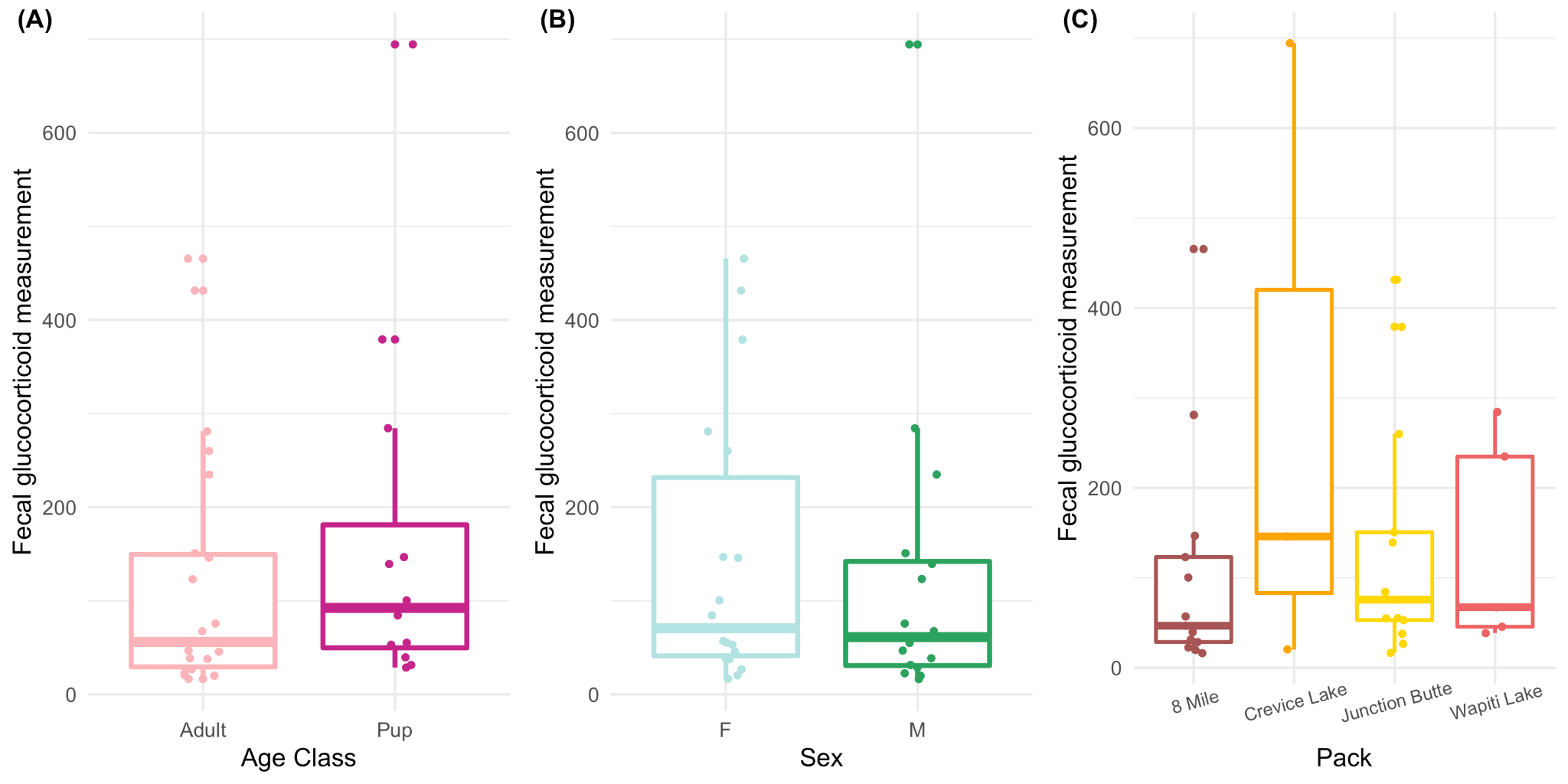
**

Figure S4. Fecal glucocorticoid measurements (ng/g of scat) stratified by wolf (A) age class, (B) sex, and (C) pack affiliation for genotyped individual wolves (n=34).

**
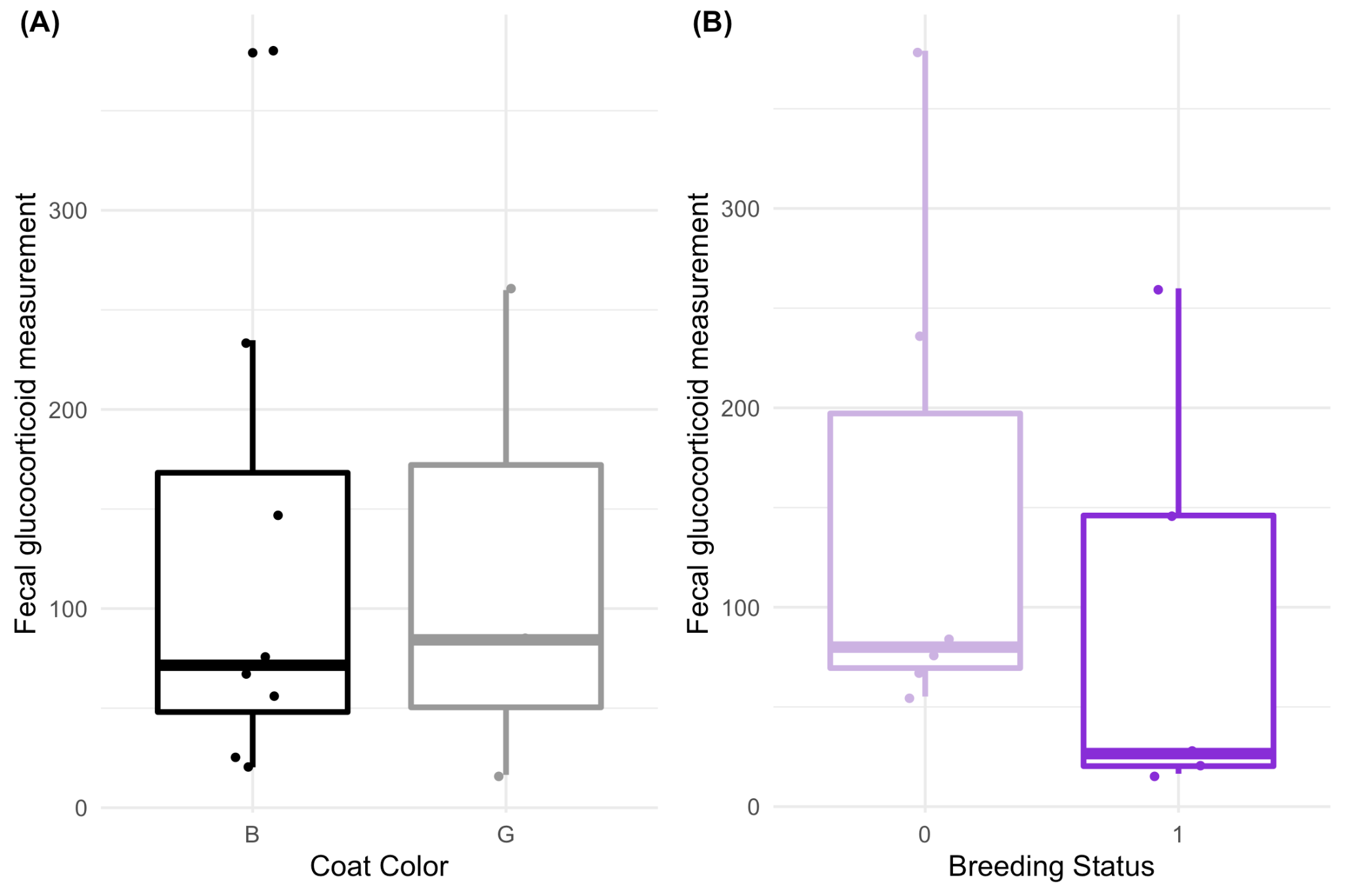
**

Figure S5. Fecal glucocorticoid measurements (ng/g of scat) stratified by wolf (A) coat color (B=black, G=gray) and (B) breeding status (0=non-breeder, 1=breeder) for collared wolves (n=11).

**SECTION IV**

Table S1. Variables considered for inclusion in the statistical models.

| **Model** | **Variable** | **Definition** | **Rationale** | **Prediction** |
| --- | --- | --- | --- | --- |
| Cestode | Age category | Adult or pup | Individuals may acquire more parasites with age because they have more time to be exposed to parasite infection [(Cattadori et al. 2005)](https://paperpile.com/c/M6UgTW/ehTkH). | Adults have higher parasite prevalence than pups. |
| Cestode | Sex | Female or male | Evidence suggests that males have higher parasite prevalence than females across many host/parasite taxa [(Poulin 1996)](https://paperpile.com/c/M6UgTW/fyeRC). | Males have higher parasite prevalence than females. |
| Cestode | Period | Summer: April-September,  Winter: October-March | Season affects parasite shedding in host scat [(Kołodziej-Sobocińska 2019)](https://paperpile.com/c/M6UgTW/E2xop). | Samples collected in winter will have higher parasite prevalence than samples collected in summer. |
| Cestode | Stress | Fecal glucocorticoid measurement (ng/g scat) | There is evidence that social status (i.e., dominance) and sex affect stress levels in wolves [(Sands and Creel 2004)](https://paperpile.com/c/M6UgTW/C3wil). Increased stress levels negatively affect immune response and the ability to fight parasite infection. | Dominant wolves have higher parasite prevalence. |
| Cestode | Pack size | Number of wolves per pack that season (maximum count, summer or winter) | Pack size influences hunting success whereby larger packs are able to kill larger prey [(MacNulty et al. 2014)](https://paperpile.com/c/M6UgTW/Q4REq), therefore smaller packs might have more diverse diets, and consequently, greater exposure.  AND/OR  Wolves in larger packs may have more support/less stress, which may increase their immune response. | Wolves in smaller packs have higher parasite prevalence. |
| Cestode | Percent elk in diet | The percent of detected kills that were elk (August 2018-June 2020) | Elk are the primary prey species for Yellowstone wolves, but as the proportion of elk in the diet decreases, wolves are consuming different species that likely harbor different cestode communities. | Wolf packs that consume less elk have higher parasite prevalence. |
| Cestode | Population density | Wolf density in northern Yellowstone that season (maximum count, summer or winter) | Environmental contamination, and therefore parasite persistence, are influenced by host population density [(Arneberg 2002)](https://paperpile.com/c/M6UgTW/KHvG2). | Parasite prevalence increases with wolf density. |
| Cestode, collared subset | Numeric age | Age in years to one decimal place; replaces *age category* | Individuals acquire more parasites with age because they have more time to be exposed to parasite infection [(Cattadori et al. 2005)](https://paperpile.com/c/M6UgTW/ehTkH). | Parasite prevalence increases with age. |
| Cestode, collared subset | Coat color | Black or gray | The k-locus confers black coat color in wolves and is linked to beta-defensin genes, which increase the responsiveness of the innate immune system [(Anderson et al. 2009)](https://paperpile.com/c/M6UgTW/xDp3e). We assume gray = missing k-locus, black = presence of k-locus. | Gray wolves have higher parasite prevalence than black wolves. |
| Cestode, collared subset | Breeding status | Breeder or non-breeder | Breeding wolves have higher stress levels [(Sands and Creel 2004)](https://paperpile.com/c/M6UgTW/C3wil). | Breeders have higher parasite prevalence than non-breeders. |
| Cestode, collared subset | Distemper | Canine distemper serological status (0/1) | Distemper is a serious acute infection that may weaken host immune responses. | Seropositive wolves have higher prevalence. |
| Cestode, collared subset | *N. caninum* | *Neospora caninum* serological status (0/1) | *N. caninum* is a chronic infection that may weaken host immune responses. | Seropositive wolves have higher prevalence. |
| Cestode, collared subset | *T. gondii* | *Toxoplasma gondii* serological status (0/1) | *T. gondii* is a chronic infection that may weaken host immune responses. | Seropositive wolves have higher prevalence. |
| Success | Days elapsed | Estimated number of days from scat deposit to collection | Unpreserved DNA molecules progressively degrade in the environment over time, and low amounts of template DNA will limit PCR success [(Lucchini et al. 2002)](https://paperpile.com/c/M6UgTW/9eLgy). | Genotyping success decreases with increasing days elapsed. |
| Success | Maximum temperature | Average daily maximum temperature from scat deposit to collection | High temperatures degrade environmental DNA and provide conditions for mold growth in scats which can also degrade DNA [(Vili et al. 2013)](https://paperpile.com/c/M6UgTW/rtd88). | Genotyping success decreases with higher average maximum temperatures. |
| Success | UV exposure | Open or covered by tree canopy/shade | Ultraviolet radiation in sunlight degrades DNA molecules [(Vili et al. 2013)](https://paperpile.com/c/M6UgTW/rtd88). We used canopy cover as a proxy for relative UV exposure. | Genotyping success is higher for samples collected under canopy cover. |
| Success | Season | Summer: April-September,  Winter: October-March,  Denning: vacated dens, mainly searched August-September | Season may account for variation in environmental factors not accounted for by other covariates. Amplification rate was higher for wolf scats collected in winter than scats collected in spring and summer for a study conducted in the Italian Alps [(Lucchini et al. 2002)](https://paperpile.com/c/M6UgTW/9eLgy). | Genotyping success is greater in winter than summer or denning. |
| Success | Precipitation | Total amount of precipitation from scat deposit to collection | Hydrolysis, or the breakage of chemical bonds through the addition of water, is one of the biggest factors affecting DNA degradation. Moisture in scats also provides conditions for mold growth which can degrade DNA [(Farrell, Roman, and Sunquist 2000)](https://paperpile.com/c/M6UgTW/2QIqv). | Genotyping success decreases with increasing precipitation. |

Three scat samples were removed from the ‘success model’ (scat 18, scat 35, scat 36) because they were found while hiking and therefore the date of deposit and pack membership could not be determined with full confidence.

**SECTION V**

[Anderson, Tovi M., Bridgett M. vonHoldt, Sophie I. Candille, Marco Musiani, Claudia Greco, Daniel R. Stahler, Douglas W. Smith, et al. 2009. “Molecular and Evolutionary History of Melanism in North American Gray Wolves.” *Science* 323 (5919): 1339–43.](http://paperpile.com/b/M6UgTW/xDp3e)

[Arneberg, Per. 2002. “Host Population Density and Body Mass as Determinants of Species Richness in Parasite Communities: Comparative Analyses of Directly Transmitted Nematodes of Mammals.” *Ecography* 25 (1): 88–94.](http://paperpile.com/b/M6UgTW/KHvG2)

[Ausband, David E., Michael S. Mitchell, Kevin Doherty, Peter Zager, Curt M. Mack, and Jim Holyan. 2010. “Surveying Predicted Rendezvous Sites to Monitor Gray Wolf Populations.” *The Journal of Wildlife Management* 74 (5): 1043–49.](http://paperpile.com/b/M6UgTW/WlSPM)

[Cattadori, I. M., B. Boag, O. N. Bjørnstad, S. J. Cornell, and P. J. Hudson. 2005. “Peak Shift and Epidemiology in a Seasonal Host–nematode System.” *Proceedings of the Royal Society B: Biological Sciences* 272 (1568): 1163–69.](http://paperpile.com/b/M6UgTW/ehTkH)

[Farrell, L. E., J. Roman, and M. E. Sunquist. 2000. “Dietary Separation of Sympatric Carnivores Identified by Molecular Analysis of Scats.” *Molecular Ecology* 9 (10): 1583–90.](http://paperpile.com/b/M6UgTW/2QIqv)

[Kołodziej-Sobocińska, Marta. 2019. “Factors Affecting the Spread of Parasites in Populations of Wild European Terrestrial Mammals.” *Mammal Research* 64 (3): 301–18.](http://paperpile.com/b/M6UgTW/E2xop)

[Lucchini, V., E. Fabbri, F. Marucco, S. Ricci, L. Boitani, and E. Randi. 2002. “Noninvasive Molecular Tracking of Colonizing Wolf (Canis Lupus) Packs in the Western Italian Alps.” *Molecular Ecology* 11 (5): 857–68.](http://paperpile.com/b/M6UgTW/9eLgy)

[MacNulty, Daniel R., Aimee Tallian, Daniel R. Stahler, and Douglas W. Smith. 2014. “Influence of Group Size on the Success of Wolves Hunting Bison.” *PloS One* 9 (11): e112884.](http://paperpile.com/b/M6UgTW/Q4REq)

[Poulin, Robert. 1996. “Sexual Inequalities in Helminth Infections: A Cost of Being a Male?” *The American Naturalist* 147 (2): 287–95.](http://paperpile.com/b/M6UgTW/fyeRC)

[Sands, Jennifer, and Scott Creel. 2004. “Social Dominance, Aggression and Faecal Glucocorticoid Levels in a Wild Population of Wolves, Canis Lupus.” *Animal Behaviour* 67 (3): 387–96.](http://paperpile.com/b/M6UgTW/C3wil)

[Stenglein, Jennifer L., Lisette P. Waits, David E. Ausband, Peter Zager, and Curt M. Mack. 2010. “Efficient, Noninvasive Genetic Sampling for Monitoring Reintroduced Wolves.” *The Journal of Wildlife Management* 74 (5): 1050–58.](http://paperpile.com/b/M6UgTW/s2MmD)

[Trejo, Bonnie S. 2012. “Comparison of Two Methods Used to Characterize the Summer Diet of Gray Wolves (Canis Lupus).”](http://paperpile.com/b/M6UgTW/pqPY7) <http://humboldt-dspace.calstate.edu/handle/2148/1031>[.](http://paperpile.com/b/M6UgTW/pqPY7)

[Vili, Nóra, Edina Nemesházi, Szilvia Kovács, Márton Horváth, Lajos Kalmár, and Krisztián Szabó. 2013. “Factors Affecting DNA Quality in Feathers Used for Non-Invasive Sampling.” *Journal of Ornithology / DO-G* 154 (2): 587–95.](http://paperpile.com/b/M6UgTW/rtd88)

[Weaver, John L., and Steven H. Fritts. 1979. “Comparison of Coyote and Wolf Scat Diameters.” *The Journal of Wildlife Management* 43 (3): 786.](http://paperpile.com/b/M6UgTW/J3HRk)
